# Risk factors for hemoglobinuria after ultrasonography-guided percutaneous microwave ablation for large hepatic cavernous hemangiomas

**DOI:** 10.18632/oncotarget.25379

**Published:** 2018-05-22

**Authors:** Fangyi Liu, Xiaoling Yu, Zhigang Cheng, Zhiyu Han, Jianping Dou, Jie Yu, Ping Liang

**Affiliations:** ^1^ Department of Interventional Ultrasound, Chinese PLA General Hospital, Beijing, 100853 China

**Keywords:** microwave ablation, hepatic cavernous hemangioma, ultrasound guidance, hemoglobinuria, risk factors

## Abstract

Thermal ablation of large hepatic cavernous hemangiomas may lead to intravascular hemolysis, hemoglobinuria, and even acute renal failure. This study aimed to identify the risk factors associated with hemoglobinuria after ultrasonography-guided percutaneous microwave ablation for large hepatic cavernous hemangiomas. In our study, 11 related risk factors were analyzed using univariate and multivariate binary logistic regression model and Receiver operating characteristic curves to determine the contribution to hemoglobinuria after microwave ablation for 49 patients with 51 hepatic cavernous hemangiomas. By multivariate analysis, the ablation time (*p* = 0.021; Odds Ratio, 1.005), and the number of antenna insertions (*p* = 0.036; Odds Ratio, 3.568) were the independent risk factors associated with hemoglobinuria. The cutoff value for ablation time and the number of antenna insertions in predicting the presence of hemoglobinuria was 1185s (sensitivity, 75%; specificity, 69%) and 4.5 (sensitivity, 55%; specificity, 83%), respectively. Less than 5 of antenna insertions and less than 20 mins of ablation time may therefore be recommended in patients with microwave ablation of large hepatic cavernous hemangiomas, in order to reduce the occurrence of hemoglobinuria. This is the first report about the risk factors analysis associated with hemoglobinuria after thermal ablation for large hepatic cavernous hemangiomas.

## INTRODUCTION

Hepatic cavernous hemangiomas (HCHs) are the most common benign neoplasms in liver, with the prevalence in the general population as many as 20% in autopsy studies [1]. Hemangiomas are referred to as “giant” if greater than 4 cm in diameter [2]. Although most hemangiomas are asymptomatic and can be managed safely with observation alone, larger lesions may produce a variety of symptoms and signs, including abdominal pain, dyspepsia, jaundice, thrombocytopenia and even spontaneous rupture [3, 4]. The primary treatment is surgical resection, transarterial embolization, radiation therapy, and the use of a vascular endothelial growth factor (VEGF) inhibitor have also been reported [5–8]. Thermal ablation, such as radiofrequency ablation (RFA) and microwave ablation (MWA), was safe, well-tolerated, and effective in markedly shrinking large HCHs and improving symptoms in most patients [9–12]. Hemoglobinuria after thermal ablation of HCHs was a common side effect and and should be taken seriously. Hemoglobinuria is a type of abnormal urine (deep-yellow-colored or wine-colored) with hemoglobin (Hb), which is often found in intravascular hemolysis leading to cell-free Hb released into circulatory system [13]. Because hemangiomas are sinusoids and the main component inside is the blood, thermal ablation by inserting the ablation needle into the tumor can lead to massive red cell destruction, intravascular hemolysis, and even acute kidney injury. Van Tilborg AA et al. reported two patients with very large symptomatic HCHs who developed acute kidney injury (AKI) shortly after bipolar RF ablation, caused by massive heat-induced intravascular hemolysis [14]. Determining risk factors associated with hemoglobinuria after thermal ablation for large hepatic cavernous hemangiomas is beneficial for reducing the risk of ablation. To our knowledge, there has been no report about the risk factors analysis associated with hemoglobinuria after thermal ablation for large hepatic cavernous hemangiomas. This study aimed to identify the risk factors associated with hemoglobinuria after ultrasonography-guided percutaneous microwave ablation for large hepatic cavernous hemangiomas.

## RESULTS

### Ablation efficacy

**Figure 1:**
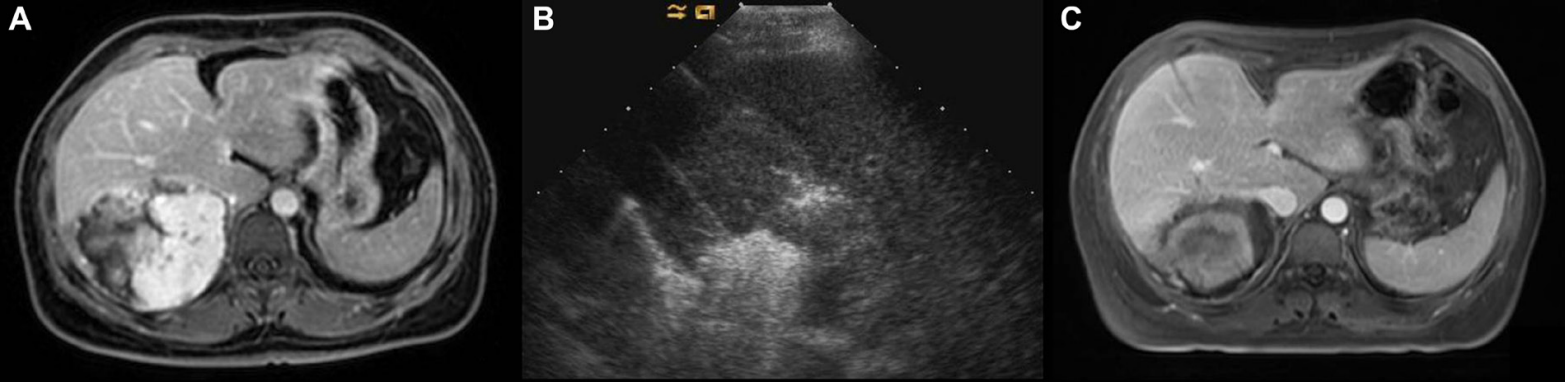
Images in a 39-year-old-woman who underwent MWA for hepatic cavernous hemangioma 10.4 cm in diameter (**A**) Preoperative contrast-enhanced MRI showed the hemangioma was hyperenhanced in portal vein phase in right lobe. (**B**) US image showed the two antennae were placed into the tumor and ablated simultaneously. (**C**) Postoperative contrast-enhanced MRI showed complete necrosis of the tumor and and the maximum diameter was reduced to 7.8 cm 3 days after MWA.

All patients were performed MWA successfully. Technical effectiveness rate was 100% with a mean ablation time of 1251.2 ± 535.1 (range 480–2730) seconds. Within 3 days after ablation, 44 lesions were necrosis completely, and 5 lesions was more than 90% necrosis due to the peripheral of the tumor adjacent to dangerous location. The mean tumor volume shrinkage rate was 53.05 ± 23.81% within 3 days after ablation. (Figure 1) Out of 20 patients who had hemoglobinuria after microwave ablation, the color of urine and urine routine test recovered normally gradually after basification of urine and hydration treatment in 19 cases. One case developed acute kidney injury (AKI) shortly after MWA, caused by massive heat-induced intravascular hemolysis. Lab results showed AKI (creatinine 227 micromol/l and urea 13.8 mmol/l in the second day after MWA, creatinine 353 micromol/l and urea 15.1 mmol/l 3 days later after MWA). Because of progressive dyspnea and ongoing anuria haemodialysis through a femoral catheter was started. After 12 hemodialysis, 32 days later, the renal function gradually recovered and dialysis was stopped and the patient was discharged from the hospital 34 days after the procedure.

The preoperative characteristics and postoperative findings of the patients are shown in Table 1. Tumor maximum diameter was significantly larger among the patients with hemoglobinuria than among those with non-hemoglobinuria after MWA (*p* = 0.007). Ablation time was significantly longer among the patients with hemoglobinuria than among those with non-hemoglobinuria after MWA (*p* = 0.004). The number of antenna insertions was significantly more among the patients with hemoglobinuria than among those with non-hemoglobinuria after MWA (*p* = 0.005). Patients with hemoglobinuria use more energy than non hemoglobinuria patients after MWA (*p* = 0.001).There were no significant differences in liver function and renal function between the two groups before MWA. There were significant differences in ALT and AST level and renal function between the two groups in the second day after MWA (*p* < 0.05).

**Table 1:** Comparison of clinical parameters between hemoglobinuria group and non-hemoglobinuria group patients after microwave ablation for large hepatic cavernous hemangiomas

Variable	hemoglobinuria (*n =* 20)	Non-hemoglobinuria (*n =* 29)	*P* value
Age (y)	43.85 ± 7.31	42.75 ± 8.89	0.653
Gender (women/men)	15/5	21/8	0.840
Max diameter (cm)	8.26 ± 1.92	6.89 ± 1.48	0.007
Ablation time (s)	1506.00 ± 440.86	1075.51 ± 529.82	0.004
Tumor volume (ml)	156.24 ± 80.89	119.62 ± 71.45	0.101
No. of antenna insertion	4.80 ± 1.32	3.72 ± 1.22	0.005
Energy (J)	154650 ± 59004	92151 ± 36367	0.001
ALT-pre (U/L)	18.55 ± 11.71	16.65 ± 12.02	0.591
AST-pre (U/L)	17.10 ± 6.54	15.75 ± 4.53	0.408
STB-pre (U/L)	13.41 ± 4.56	11.75 ± 3.97	0.195
ALP-pre (U/L)	60.77 ± 14.26	58.20 ± 11.10	0.495
Cre-pre (μmol/L)	67.81 ± 12.42	63.60 ± 11.56	0.239
BUN-pre (mmol/L)	4.70 ± 1.25	4.36 ± 1.13	0.329
ALT-post (U/L)	216.42 ± 189.54	100.22 ± 61.70	0.003
AST-post (U/L)	313.71 ± 281.30	145.73 ± 116.43	0.006
STB-post (U/L)	41.07 ± 31.86	27.37 ± 16.31	0.054
ALP-post (U/L)	56.52 ± 12.99	57.45 ± 12.45	0.823
Cre-post (μmol/L)	90.08 ± 47.12	59.87 ± 17.04	0.004
BUN-post (mmol/L)	6.95 ± 6.04	3.82 ± 1.59	0.013
Tumor reduction rate (%)	52.14 ± 25.35	53.97 ± 22.97	0.832

Abbreviations; ALT: alanine aminotransferase; AST: aspartate aminotransferase; STB: total bilirubin; ALP: alkaline phosphatase; Cre: creatinine; BUN: blood urea nitrogen; ^*^-pre: index of preablation; ^*^-post: index of postablation.

### Risk factors of hemoglobinuria

By univariate analysis, tumor maximum diameter (*p* = 0.017), ablation time (*p* = 0.01), and the number of antenna insertions (*p* = 0.01) were statistically significant risk factors after MWA for HCHs. By multivariate analysis, the ablation time (*p* = 0.021; Odds Ratio, 1.005), and the number of antenna insertions (*p* = 0.036; Odds Ratio, 3.568) were the independent risk factors associated with hemoglobinuria after MWA for HCHs. (Table 2) When analyzed with use of ROC curves, ablation time (AUC, 0.759; 95% CI: 0.626, 0.892; *P* = 0.002) and the number of antenna insertions (AUC, 0.722; 95% CI: 0.573, 0.870; *P* = 0.009) were also significant factors for predicting hemoglobinuria. The cutoff value for ablation time in predicting the presence of hemoglobinuria was 1185s (sensitivity, 75%; specificity, 69%). The cutoff value for the number of antenna insertions in predicting the presence of hemoglobinuria was 4.5 (sensitivity, 55%; specificity, 83%) (Table 3).

**Table 2:** Univariate and multivariate analysis of clinical parameters related to hemoglobinuria after microwave ablation for large hepatic cavernous hemangiomas

Factor	Univariate analysis		Multivariate analysis	
	*P* Value	Odds Ratio	*P* Value	Odds Ratio
Age	0.645	1.017	0.872	0.987
Gender	0.840	1.143	0.093	27.720
Max diameter	0.017	1.668	0.860	1.104
Tumor volume	0.108	1.007	0.424	1.011
ALT level	0.584	1.014	0.155	1.171
AST level	0.408	1.048	0.906	0.970
STB level	0.197	1.100	0.320	1.196
BUN level	0.323	1.286	0.498	1.505
Cre level	0.236	1.031	0.126	1.126
Ablation time	0.010	1.002	0.021	1.005
NO. of antenna insertion	0.010	1.971	0.036	3.568

Abbreviations; ALT: alanine aminotransferase; AST: aspartate aminotransferase; STB: total bilirubin; ALP: alkaline phosphatase; Cre: creatinine; BUN: blood urea nitrogen.

**Table 3:** Sensitivity, specificity, and cutoff values for risk factors in predicting the presence of hemoglobinuria after microwave ablation for large hepatic cavernous hemangiomas

Factors	Cutoff value	Sensitivity (%)	Specificity (%)
Ablation time	1185s	75	69
No. of antenna insertions	4.5	55	83

## DISCUSSION

For most patients with HCHs, open or laparoscopic surgical resection has been considered the preferred choice of treatment [17]. However, it is important to keep in mind that many of the trade-offs encountered during the treatment of cancer are not applicable to HCHs due to their benign natural history. Thus, a highly effective but morbid treatment would be a poor choice for most patients. Percutaneous thermal ablation, such as RFA and MWA, was safe, well-tolerated, and effective treatment method for most patients with HCHs [9–12]. However, due to the characteristics of the hemangioma itself, thermal ablation for hemangiomas may lead to a mass of red blood cells undergoing budding and fragmentation, which presumably resulted in a massive heat-induced intravascular hemolysis. Massive hemolysis can lead to various degrees of hemoglobinuria, hemolytic jaundice, anemia, and renal damage [11]. Determining risk factors associated with hemoglobinuria after thermal ablation for large HCHs is beneficial for reducing the risk of ablation, while there have been no report about it.

In our study, tumor maximum diameter, ablation time and the number of antenna insertions were statistically significant risk factors by univariate analysis. By multivariate analysis, we discovered that long ablation time and more antenna insertions during MWA can lead to hemoglobinuria with statistically significant cutoff values of 1185s (nearly 20 mins) and 4.5, respectively. Therefore, when treating large HCHs with MWA, the number of antenna insertions and the ablation time should be reduced to the absolute minimum, in order to prevent hemoglobinuria. Van Tilborg AA et al. reported two patients with very large symptomatic HCHs who occurred hemoglobinuria and developed acute kidney injury shortly after bipolar RF ablation, and thought it was related with the size of the ablation zone and length of the procedure [14]. The goal of MWA for hemangioma and other benign lesions is tumor conformal necrosis or most part necrosis, so the ablation zone is basically the same as the tumor volume, but tumor volume were not statistically significant risk factors by univariate analysis, while the maximum tumor diameter is statistically significant only in univariate analysis, not in multivariate analysis in our study. During thermal ablation for HCHs, hemoglobin is released upon erythrocyte destruction and is filtered by the glomerulus into the urinary space. In the urinary space, hemoglobin is degraded and releases hemepigments which are toxic to the kidney. Hemepigments can cause tubular injury by (1) tubular obstruction, (2) damage due to direct proximal tubular cell injury, and (3) vasoconstriction, resulting in reduced blood flow in the outer medulla [18]. So, proper treatments including adequate hydration to decrease the Hb concentration in circulation system, alkalization of urine to make Hb crystal dissolution and prevent tubular obstruction and even acute kidney injury.

This study had some limitations. First, hemoglobinuria was qualitatively determined by urine routine without quantitative analysis. Second, the present study was a retrospective analysis which was performed in a single institution. The results must be confirmed in another cohort or in a prospective multicenter-study.

## MATERIALS AND METHODS

### Patients

From January 2011 to December 2016, 49 patients (36 females, 13 male; average age 43.20 ± 8.22 years) with 51 giant hepatic hemangiomas (mean maximum diameter 7.45 ± 1.78 cm, range 4.1–12.6 cm) treated with image-guided percutaneous MWA were reviewed in this study. Among them, 20 patients had hemoglobinuria confirmed by urine routine after MWA. The patients were divided into two groups: hemoglobinuria group and non-hemoglobinuria group based on whether hemoglobinuria occurred after MWA.

Inclusion criteria for performing MWA were (1) definite diagnosis of a giant cavernous hemangioma > 4 cm based on the typical enhancement pattern on contrast-enhanced multiphase computed tomography (CT) or magnetic resonance imaging (MRI); (2) clinical symptoms typically caused by the giant hemangioma present for at least 1 year, including abdominal pain, nausea, vomiting, abdominal fullness. The diagnoses of HCHs were proven pathologically in all patients using US-guided core needle biopsy followed by ablation. This clinical application was approved by our institutional human research review committee. Written informed consent was obtained from all patients.

### Microwave equipment and ablation technique

All treatments were performed in our institution and were carried out under US guidance with the patients under unconscious intravenous anesthesia (Propofol, 6–12 mg/kg/h; Ketamine, 1–2 mg/kg) in the operating room. The 2450 MHz or 915 MHz MW system was used. The 2450 MHz MW system (KY-2000, Kangyou Medical, China) consists of three independent MW generators, three flexible coaxial cables and three water-pumping machines, which can drive three 15-gauge cooled-shaft antennae (1.1 cm antenna tip) simultaneously. The 915MHz MW system (KY-2001, Kangyou Medical, China) consists of two independent MW generators, two flexible coaxial cables and two water-pumping machines, which can drive two 15-gauge cooled-shaft antennae (2.2 cm antenna tip) simultaneously. The two MW system generators are capable of producing 1–100 W of power output. All therapy was performed by two experienced radiologists according to the preoperative planning. Hydrodissection technique and thermal monitoring technique were applied for hemangiomas abutting vital structures to avoid thermal damage. The detailed procedures of ultrasound-guided MWA in patients with liver tumors were described in our previous publications [15, 16].

### Evaluation methods

The patients’ blood tests (hepatic and renal function) and urine routine were taken before and after MWA. In the urine routine, dry chemistry analysis was used to detect Hb and flow cytometry was used to detect the red blood cells (RBCs). Hemoglobinuria was determined with the results of Hb positive and RBCs negative [13]. The wine-colored hemoglobinuria can be visually observed while deep-yellow-colored hemoglobinuria only can be detected by urine routine. Once abnormal results were discovered after MWA, hepatic or renal functions were tested every other day, and urine routine would be tested every time the patient urinated until all the results were normal. The data of tumor volume, ablation energy and ablation time in this study were all from one-session MWA. The tumor volume was calculated by the equation V = π*a*b*c/6, where a, b and c were three dimensions of the tumor measured by contrast-enhanced ultrasound. The number of antenna insertions was defined as the total number of antenna placements for each patient during ablation.

### Statistical analysis

Data analysis was performed using SPSS17.0 for windows (SPSS Inc, Chicago, IL, USA) and the continuous data were expressed as means ± standard deviations (SD). Data were analyzed between the hemoglobinuria group and non-hemoglobinuria group by using the Student *t* test for unpaired data and the x^2^ test and Fisher exact test as appropriate. 11 related risk factors, including gender, age, tumor maximum diameter, tumor volume, ablation time, the number of antenna insertions, alanine aminotransferase (ALT), aspartate aminotransferase (AST), total bilirubin (STB), blood urea nitrogen (BUN) and creatinine (Crea) were analyzed using univariate and multivariate binary logistic regression model method. Receiver operating characteristic (ROC) curves were constructed, and the area under the ROC curve (AUC) was calculated by using the trapezoidal rule. Optimal cutoff values for risk factors were selected to maximize sensitivity, specificity. All of the statistical tests were two-sided, and significance was set at *p* < 0.05.

## CONCLUSIONS

In conclusion, the ablation time and No. of antenna insertion were the independent risk factors associated with hemoglobinuria when MWA for large HCHs. Less than 5 of antenna insertions and less than 20 mins of ablation time may therefore be recommended in patients with MWA of large HCHs, in order to reduce the occurrence of hemoglobinuria.

## Abbreviations

HCHs: hepatic cavernous hemangiomas
MWA: microwave ablation
ROC: receiver operating characteristic
VEGF: vascular endothelial growth factor
RFA: radiofrequency ablation
AKI: acute kidney injury
